# Proteomics profiling and pathway analysis of hippocampal aging in rhesus monkeys

**DOI:** 10.1186/s12868-020-0550-4

**Published:** 2020-01-15

**Authors:** Shu Meng, Wenchao Xia, Meng Pan, Yangjie Jia, Zhanlong He, Wei Ge

**Affiliations:** 1 State Key Laboratory of Medical Molecular Biology & Department of Immunology, Institute of Basic Medical Sciences Chinese Academy of Medical Sciences, School of Basic Medicine Peking Union Medical College, Dongdan Santiao 5# Dongcheng District, Beijing, 100005 China; 2 Yunnan Key Laboratory of Vaccine Research and Development on Severe Infectious Disease, Institute of Medical Biology, Chinese Academy of Medical Sciences and Peking Union Medical College, Kunming, 650118 Yunnan China; 3 Department of Neurosurgery, Affiliated Hospital of Hebei University, Baoding, 071000 China

**Keywords:** Aging, Proteomics, Hippocampus, Rhesus monkey, Neurodegeneration

## Abstract

**Background:**

Aged rhesus monkeys exhibit deficits in memory mediated by the hippocampus. Although extensive research has been carried out on the characteristics of human hippocampal aging, there is still very little scientific understanding of the changes associated with hippocampal aging in rhesus monkeys. To explore the proteomics profiling and pathway-related changes in the rhesus hippocampus during the aging process, we conducted a high throughput quantitative proteomics analysis of hippocampal samples from two groups of rhesus macaques aged 6 years and 20 years, using 2-plex tandem mass tag (TMT) labeling. In addition, we used a comprehensive bioinformatics analysis approach to investigate the enriched signaling pathways of differentially expressed proteins (the ratios of 20-years vs. 6-years, ≥ 1.20 or ≤ 0.83).

**Results:**

In total, 3260 proteins were identified with a high level of confidence in rhesus hippocampus. We found 367 differentially expressed proteins related to rhesus hippocampus aging. Based on biological pathway analysis, we found these aging-related proteins were predominantly enriched in the electron transport chain, NRF2 pathway, focal adhesion–PI3K–AKT–mTOR signaling pathway and cytoplasmic ribosome proteins. Data are available via ProteomeXchange with identifier PXD011398.

**Conclusion:**

This study provides a detail description of the proteomics profile related to rhesus hippocampal aging. These findings should make an important contribution to further mechanistic studies, marker selection and drug development for the prevention and treatment of aging or age-related neurodegeneration.

## Background

Rhesus macaques are a nonhuman primate species that is closely related to humans and share a common ancestor that lived over 25 million years ago [1–3]. Rhesus macaques are also one of the most commonly used animal models in basic and applied biomedical research on human disease [1, 2]. Previous research has also established that rhesus macaques and humans share approximately 95% genetic homology and have very similar age-associated conditions including diabetes, osteoarthritis, endometriosis, visual accommodation, hypertension, osteoporosis, and amyloidosis [2]. The brain architecture of the rhesus macaque is also very similar to that in humans and displays comparable age-related cognitive decline [2–4]. Investigating the molecular mechanism of the normal aging of rhesus brain has received considerable critical attention.

The hippocampus plays a pivotal role in cerebral learning and memory and is particularly susceptible to normal aging [5]. Short-term memory is thought to depend on the hippocampus and/or amygdala in the medial temporal lobe [6, 7]. It has previously been observed that aged monkeys suffer from a profound and specific impairment in short-term memory and show a delayed-response deficit [8]. In addition, aged monkeys exhibit impaired recognition memory [9]. However, the volume of the hippocampus (total or any of its subfields) remains stable in rhesus monkeys during aging [10–12]. This is in contrast to a substantial number of MRI studies showing a reliable age-related decline in hippocampal volume in healthy humans [10]. Aged monkeys have been reported to have significantly lower regional cerebral blood flow (rCBF) and reduced regional cerebral metabolic rate for glucose (rCMRglc) in the hippocampus [13]. However, the basic mechanisms of rhesus hippocampal aging and cognitive decline have never been elucidated.

It is now generally accepted that brain aging is accompanied by changes in protein profiles, which lead to impairment of key biological pathways. Proteomics and genomics studies provide complementary evidence. The application of quantitative proteomics using tandem mass tag (TMT) labels has facilitated the use of proteomic approaches in various areas of research, including neurological studies of aging [5].

Following our previous proteomics study of human hippocampal aging [14], we conducted a 2-plex TMT proteomics analysis of the hippocampus in two groups of rhesus macaques with mean age of 6 years and 20 years respectively. We also conducted a bioinformatics analysis of the aging-related proteins identified in the hippocampus, with the aim of profiling differentially expressed proteins and identifying possibly altered signaling pathways during aging.

## Results

### High-confidence proteins

**Fig. 1:**
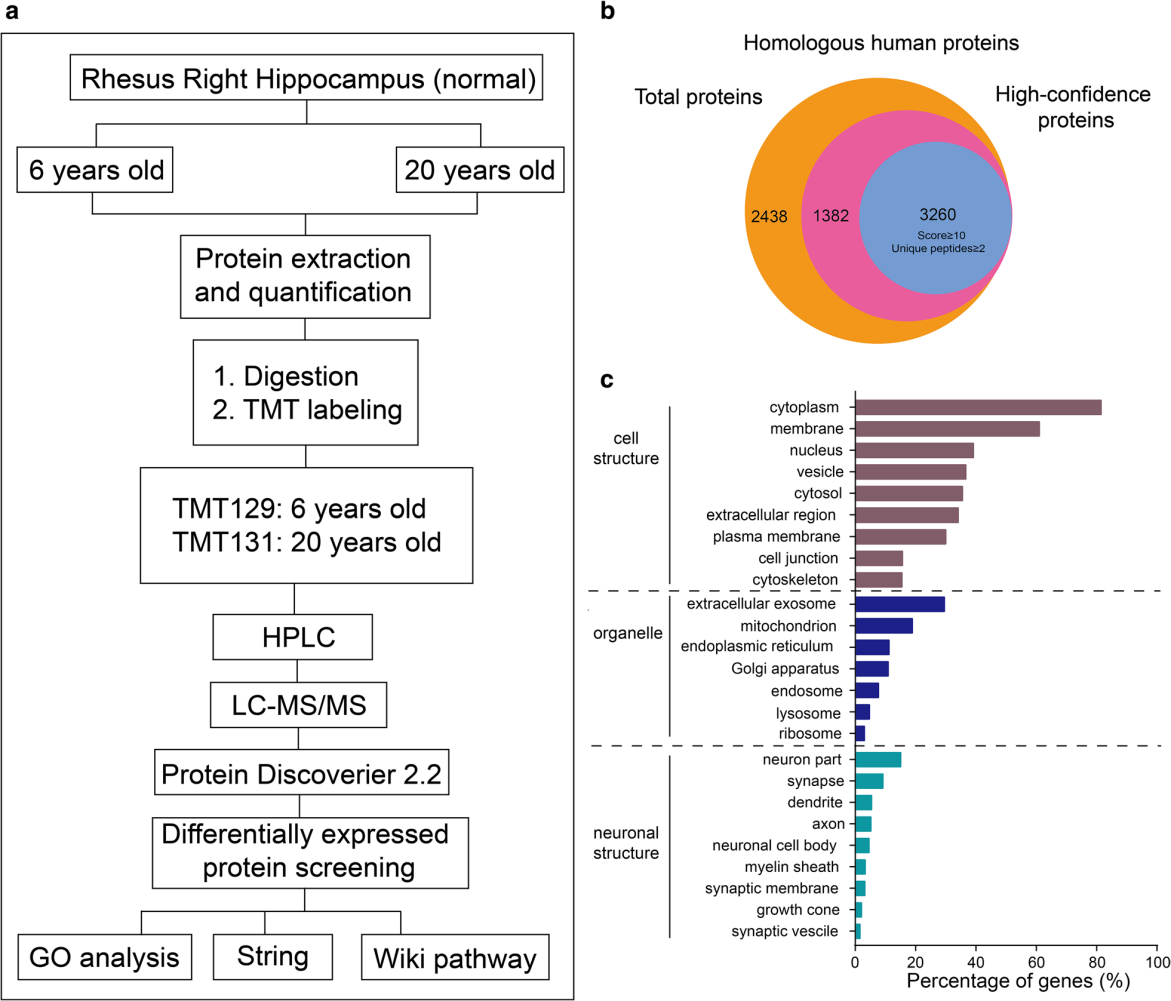
The workflow and characteristics of high-confidence proteins identified during rhesus hippocampal aging. **a** The workflow of the proteomics analysis. **b** The homology analysis and high-confidence protein selection. **c** Gene ontology classification of the high-confidence proteins. The percentage of proteins enriched in each category is indicated

In the LC–MS/MS analysis, a total of 7080 rhesus proteins were identified, of which 4642 proteins were human protein homologs and 3260 were identified by two or more peptides with scores ≥ 10 (Fig. 1b, Additional file 1: Table S1). The GO classification of all identified high-confidence proteins was analyzed using DAVID. To assess the proteome composition, we annotated all the identified proteins to cellular component (CC) gene ontology (GO) terms (Fig. 1c). The largest proportion of proteins (81.2%) was annotated as “cytoplasmic” with a slight preferential distribution in “extracellular exosome” (29.5%), “mitochondrion” (18.9%), and “endoplasmic reticulum” (11.2%). Approximately 24.4% of annotated proteins corresponded to neuron-specific GO terms such as “synapse” (9.2%), “dendrite” (5.4%), “axon” (5.2%), and “neuronal cell body” (4.6%).

### Screen and GO analysis of aging-related proteins in the rhesus hippocampus

**Fig. 2:**
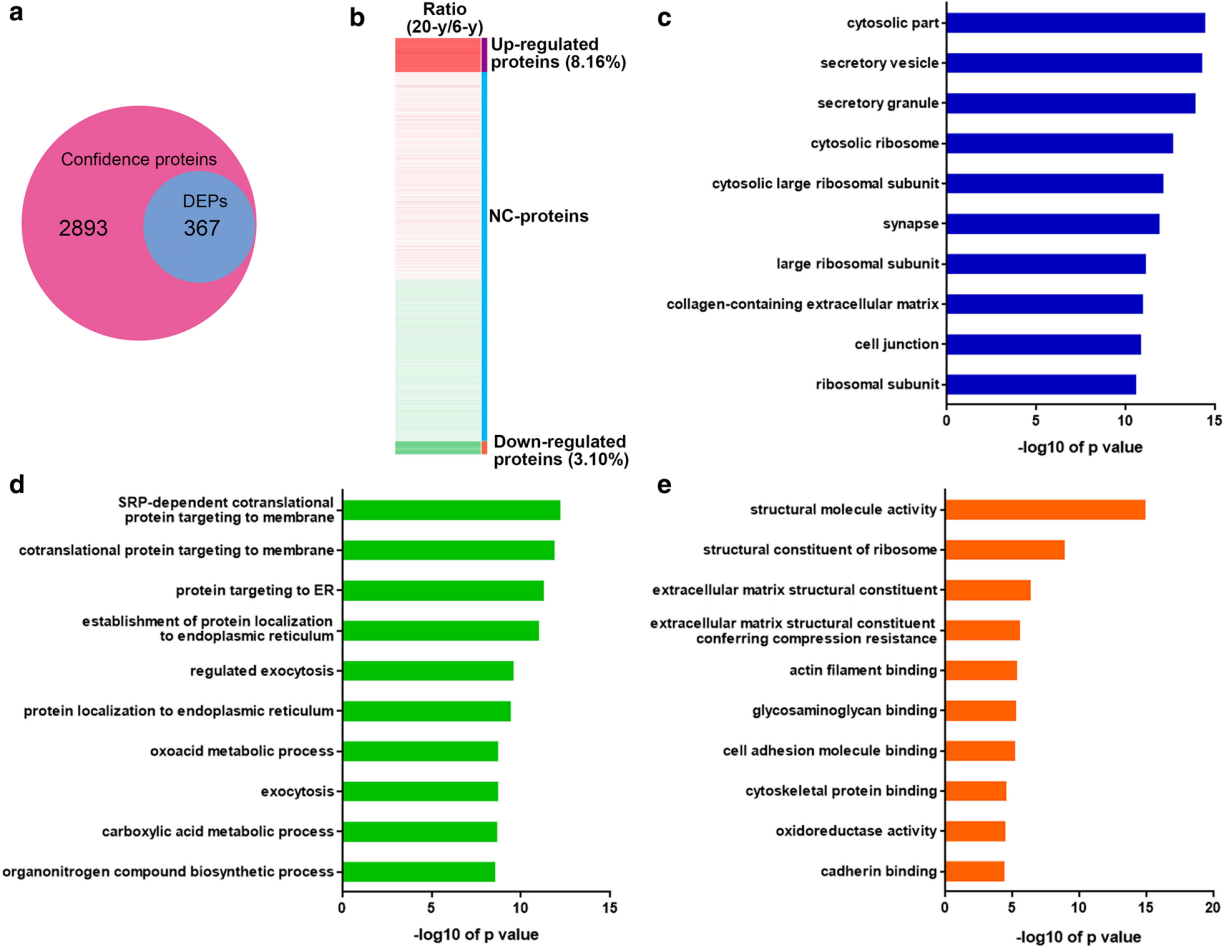
Profiling of differentially expressed proteins (DEPs) during rhesus hippocampal aging. **a** Venn diagram showing the selection of DEPs with thresholds of ≥ 1.20 or ≤ 0.83 (20-year vs. 6-year). **b** Heatmap analysis of DEPs. NC-proteins, non significantly changed proteins. **c**–**e** The gene ontology categories of DEPs in cellular components (**c**), biological process (**d**), and molecular functions (**e**). The enrichment p value in each category is indicated

As shown in Fig. 2a, the ratios of proteins from 20-year old rhesus vs 6-year old rhesus were used to evaluate the expression levels of proteins quantitatively. Using threshold values of ≥ 1.20 for upregulation and ≤ 0.83 for downregulation of proteins, we found 367 differentially expressed proteins (DEPs) were related to hippocampal aging (Fig. 2a), including 266 (8.16%) upregulated proteins and 101 (3.10%) downregulated proteins (Fig. 2b). The top 30 significantly upregulated and downregulated proteins identified in comparisons of the samples from macaques (aged 20 years) with macaques (aged 6 years) are shown in Additional file 1: Table S1.

The enriched GO terms of aging-related proteins of rhesus hippocampus were predicted using the WEB-based GEne SeT AnaLysis Toolkit. For cellular component, the DEPs of hippocampus were mainly enriched in cytosolic part, secretory vesicle and granule, and cytosolic ribosomes (Fig. 2c). For biological processes, the DEPs associated with hippocampal aging were primarily enriched in cotranslational protein targeting to membrane, protein targeting to ER, and establishment of protein localization to ER (Fig. 2d). In addition, the most important molecular functions of the DEPs were structural molecule activity (Fig. 2e).

**Fig. 3:**
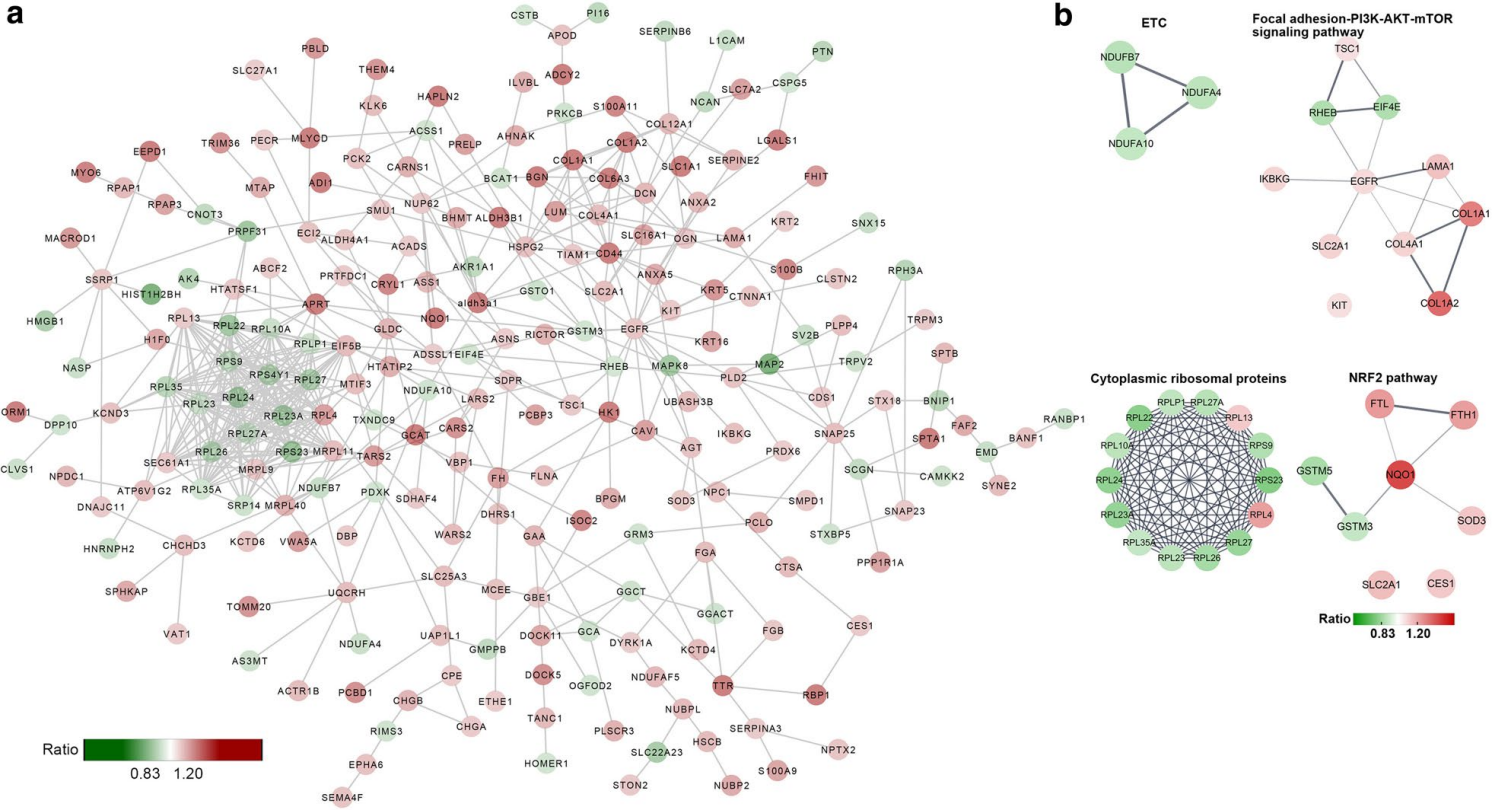
The STRING analysis of the different expression proteins (DEPs) of rhesus hippocampal aging. **a** All DEPs were analyzed by cytoscape for protein interactions. **b** String analysis for DEPs enriched in pathways. Proteins in the networks are shown as nodes. The direction and degree of the change (Abundance ratio) in expression is indicated by the color (upregulated in red and downregulated in green)

To gain a better understanding of the protein–protein interactions among the DEPs identified during normal aging of the rhesus hippocampus, we utilized the Cytscape 3.7.0 to create comprehensive networks according to the same criteria used in the pathway analysis (≤ 0.83- or ≥ 1.20-fold). For proteins interactions, minimum required interaction score was set with medium confidence (0.4), and the maximum additional interactors were set at 0. In the networks, we used shortened protein names, and grouped proteins with similar functions or belonging to the same protein families. Most of the proteins showed close interactions with each other. Interestingly, a group of ribosome-related proteins (RPL22, RPL10A, RPS9, RPS4Y1, RPL27, RPL35, RPL23, RPL24, RPL23A, RPL27, RPL27A, RPL26, RPS23) with close interactions were found in the network, indicating protein synthesis is under tight regulation during aging (Fig. 3a).

### Pathway analysis of aging-related proteins of the rhesus hippocampus

**Fig. 4:**
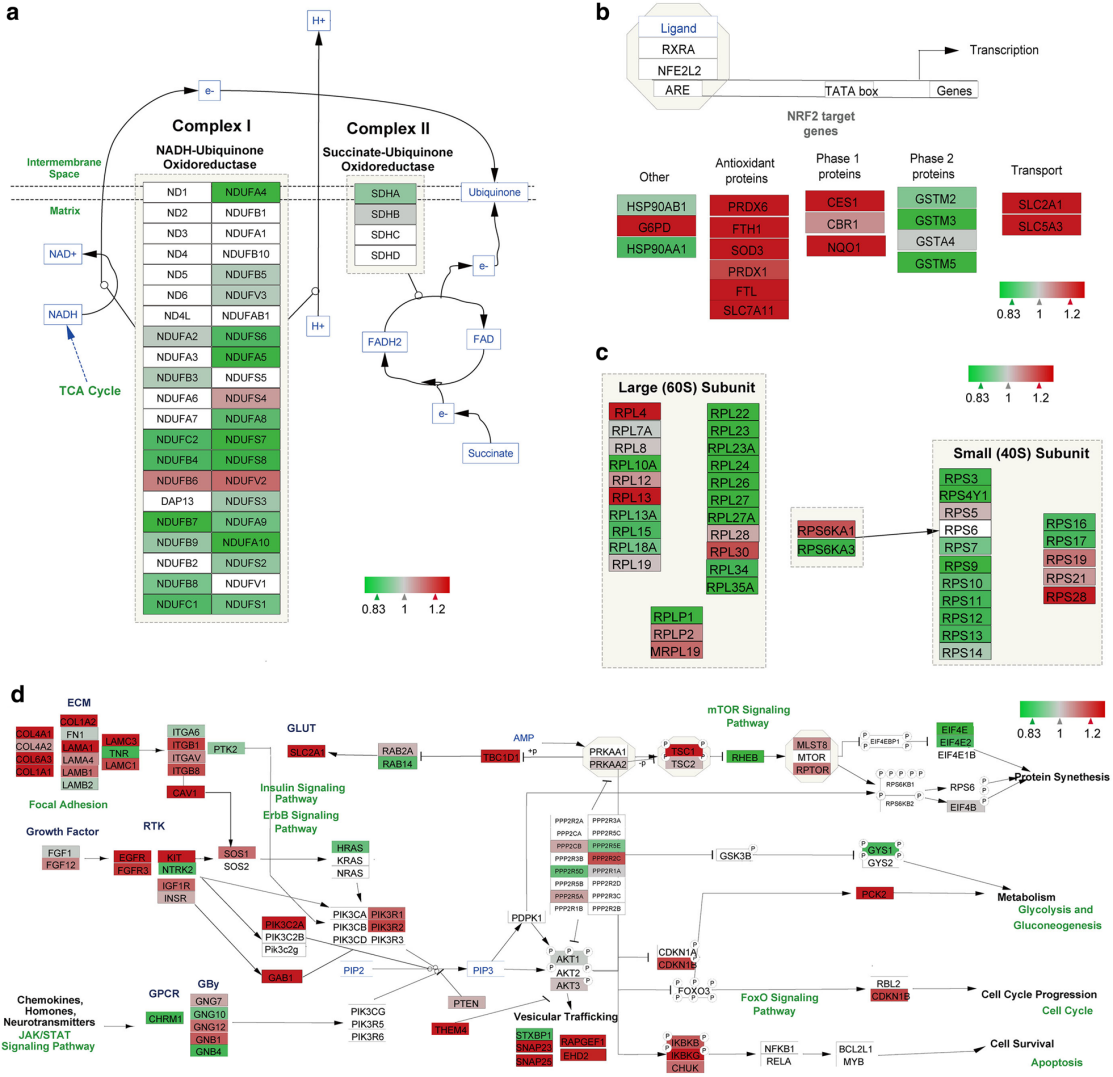
Wiki pathway analysis of the differentially expressed proteins (DEPs) during rhesus hippocampal aging. The DEPs predominantly enriched in electron transport chain (**a**), NRF2 pathway (**b**), cytoplasmic ribosome proteins (**c**), and focal adhesion PI3K–AKT–mTOR signaling pathway. The upregulated proteins are shown in red and the downregulated proteins are shown in green

To provide insights into the biological pathways associated with the DEPs during aging of hippocampus, the WEB-based GEne SeT AnaLysis Toolkit (http://www.webgestalt.org/option.php) was employed to map the gene symbol of the DEPs to the Wiki pathway database. Cytoscape software (version 3.6.0) was used to visualize these differentially expressed proteins in matched pathways. The results indicated that hippocampal aging-related proteins were predominantly involved in 9 pathways, including the electron transport chain (ETC), NRF2 pathway, cytoplasmic ribosomal proteins, and the focal adhesion–PI3K–AKT–mTOR signaling pathway (Additional file 2: Table S2). Protein–protein interaction networks of DEPs detected in these pathways were visualized by cytoscape, and close interactions were found for these DEPs (Fig. 3b). Proteins in ETC were markedly influenced with rhesus hippocampal aging (Fig. 4a). Almost all proteins in complex I were downregulated in the hippocampus during aging from 6 to 20 years. Among these proteins, NDUFA4, NDUFA5, NDUFS7, NDUFS8, NDUFB7, and NDUFA10, are more sensitive to aging than other proteins. ETC dysfunction in the mitochondria has a great impact on oxidative phosphorylation and ATP synthase activity and induces the upregulation of reactive oxygen species (ROS). To combat to the oxidative damage caused by ROS, the NRF2 pathway was activated to produce antioxidant proteins, such as PRDX6, FTH1, SOD3, FTL, and SLC7A11 (Fig. 4b). Subsequently, differential expression (up- and downregulation) of several downstream NRF2 pathway proteins, such as G6PD, NQO1, CES1, SLC2A1, SLC5A3, GSTM3, and GSTM5, were detected. Furthermore, the results also displayed widespread downregulation of ribosome proteins including RPL22, RPL23A, RPL10A and so on (Fig. 4c). During aging of the hippocampus, several aging-related extra- and intra-signaling transduction were identified, such as focal adhesion signaling, insulin signaling, ErbB signaling, mTOR signaling, FoxO signaling, and apoptosis. In particular, we observed marked activation of the focal adhesion–PI3K–AKT–mTOR signaling pathway (Fig. 4d). This was reflected by upregulation of collagen (COL4A1, COL6A3, COL1A1, COL1A2), integrin (ITGB1, ITGB8), and CAV1. In addition, we observed upregulation of TSC1 and downregulation of RHEB, EIF4E, and EIF4E2 in the mTOR signaling pathway during hippocampus aging.

### Verification of protein expression levels

**Fig. 5:**
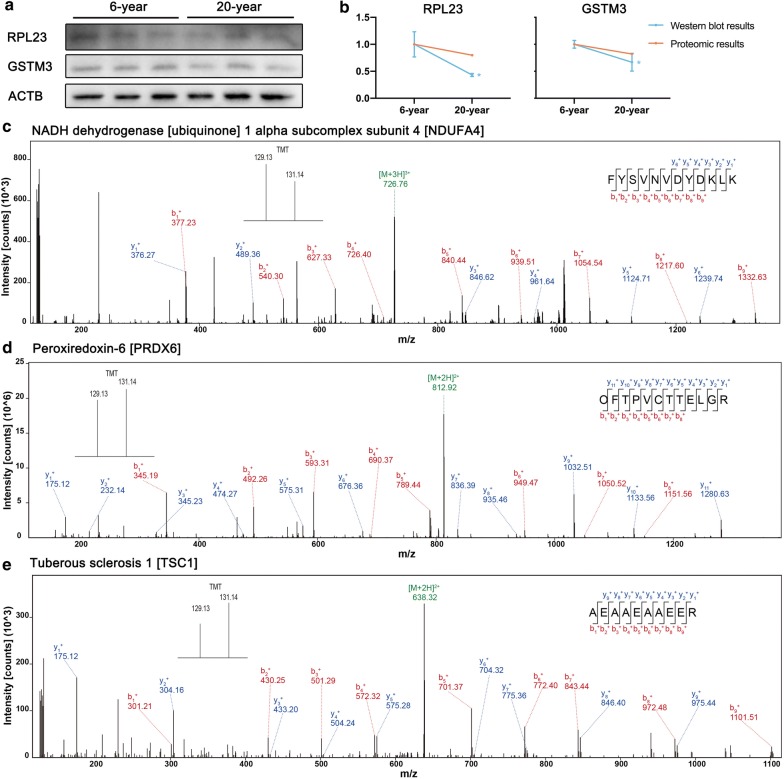
The changes in protein expression were confirmed by Western blot and MS/MS analyses. **a** Western blot analysis of GSTM3, RPL23 and ACTB proteins. **b** The results were consistent with the proteomics data. *p < 0.05 (two-tailed Students’ *t* test). **c**–**e** The representative MS/MS spectrum data of NDUFA4, PRDX6, TSC1. The column height of the TMT diagram indicates the relative quantification of the peptide segment in 6-year group and 20-year group. 129.13 (TMT-129) for 6-year group, 131.14 (TMT-131) for 20-year group

To validate the reliability of the quantitative proteomic analysis, the individual protein sample from 6-year and 20-year groups was subjected to Western blot analysis. Two proteins (RPL23, GSTM3) were identified in this study. There was a high level of similarity in the binding sequences of the anti-RPL23, anti-GSTM3 and anti-ACTB antibodies for human and rhesus macaque RPL23, GSTM3 and ACTB (Additional file 3: Figure S1). Significant downregulation of RPL23 and GSTM3 (20-year/6-year) were found with increasing age (p < 0.05). The fluctuation in the levels of these proteins was consistent with those observed in the proteomics data (Fig. 5a, b). The representative MS/MS spectrum data for NDUFA4, PRDX6, TSC1 are displayed for validation of the proteomics data in Figs. 5c–e.

## Discussion

Gerontological studies in rhesus monkeys can help identify possible mechanisms of aging and age-related chronic diseases and evaluate possible interventions with potential relevance to human aging and disease. However, much of the aging-related research to date has been conducted in human and mouse models, and the molecular changes associated with aging in rhesus monkeys have not been widely investigated. To our knowledge, this is the first study to elucidate protein changes and pathways related to rhesus hippocampal aging and is the only rhesus monkey hippocampal proteome database published to date (Additional file 2: Table S2).

The hippocampus plays a critical role in learning and memory. Given its major involvement in brain aging and neurodegenerative diseases, this study was designed to determine aging-associated changes in protein expression in the hippocampus. Studies have shown marked impairment of short-term and recognition memory in elderly rhesus monkeys (≥ 18 years and 25–27 years, respectively) compared to their younger counterparts (aged 3–5 years and 5–7 years, respectively) [8, 9]. To confirm that the protein changes are primarily age-related, we investigated protein expression in hippocampal tissues selected young and old rhesus monkeys aged 6 and 20 years, respectively.

Based on high throughput TMT-labeled quantitative proteomics analysis, the variations in protein expression associated with rhesus hippocampal aging (from 6 to 20 years old) were evaluated in current study. A total of 3260 confident hippocampal proteins were identified by high resolution mass spectrometry, including 367 DEPs associated with rhesus hippocampal aging. Our pathway analysis results showed that ETC, the NRF2 pathway, cytoplasmic ribosomal pathway, and the focal adhesion–PI3K–AKT–mTOR signaling pathway were particularly related to rhesus hippocampal aging.

Recent genomic studies suggest that, transcriptionally, components of ETC are particularly affected by aging. One comparative study of the microarray data between *Caenorhabditis elegans* and *Drosophila* during the aging process revealed a small (approximately twofold) decrease in a large set genes involved in ATP synthesis and mitochondrial respiration in both species [15]. The link between mitochondrial metabolism and longevity is also supported by several studies demonstrating that direct disruption of the ETC, such as *isp*-*1,* and various components of respiratory chain complexes I, III, IV, or V can have a significant effect on lifespan [16–18]. A decrease in electron transport activity in mitochondria isolated from rhesus brain is well documented. Complex I, in particular, and complex IV show reduced enzymatic activity in mitochondria [19]. The association between ETC, particularly complex I, and aging has been reported frequently and the primary cause of aging was suggested to be mitochondrial decline [20]. In addition, recent studies indicated that respiratory complex I induced increased ROS production resulting in mitochondrial damage and furthermore, that inhibition of respiratory complex I can extend the lifespan of *Drosophila* [21]. The generation of ROS is increasingly recognized to play an important role in both aging and neurodegenerative diseases. ROS are considered the major cause of aging because they damage proteins, lipids, and DNA by oxidation [22]. The marked downregulation of ETC proteins was also verified in older humans with impaired glucose tolerance and in a mouse model of impaired glucose tolerance [23, 24]. In our study, the marked downregulation of complex I proteins was also revealed in rhesus hippocampus during aging, indicating a role in aging of rhesus hippocampus.

Oncogene-induced NRF2 transcription has been shown to induce the expression of a variety of proteins with detoxification and antioxidative functions that protect against ROS-induced damage [25–28]. Studies of the rat hippocampus also showed that allicin significantly ameliorated aging-induced cognitive dysfunction through enhancing of Nrf2 antioxidant signaling [29, 30]. The role of the NRF2 pathway in rhesus hippocampal aging has not yet been explored. According to our data, obvious upregulation of downstream proteins, such as PRDX6, SLC7A11, SOD3, G6PD and NQO1, were detected in the NRF2 pathway. PRDX6, SLC7A11, and SOD3 are characteristic antioxidant proteins of the NRF2 pathway and play a significant role in the aging process. PRDX6 reduces H_2_O_2_ to H_2_O in a reaction catalyzed by thioredoxin and reduces lipid hydroperoxides to corresponding alcohols by GSH [31]. Age-related reduction in PRDX6 expression in trabecular meshwork cells has been shown to delay senescence by protecting against the damage induced by ROS accumulation during the aging process [32]. In accordance with the findings in aged trabecular meshwork, the age-related upregulation of PRDX6 in rhesus hippocampus suggests an efficient PRDX6 repair mechanism during rhesus hippocampal aging. In addition, SOD3 maintains corneal endothelial integrity during aging and the SOD3 R213G polymorphism caused premature aging of transgenic mice by inducing inflammation through ROS-mediated damage [33, 34]. It was reported that the basal expression of SOD3 changes with aging in a tissue-specific manner. In the prostatic lobes and renal cortex of rats, SOD3 expression was increased with aging, while expression was decreased in retinal pigment epithelial cells [31]. SOD3 expression in hippocampal aging in rhesus monkeys has not yet been investigated. In this study, we revealed increased basal expression of SOD3 in rhesus hippocampal aging from 6 to 20 years. Furthermore, the NQO1 C609T polymorphism is frequently associated with various age-related pathologies and has been shown to impair the age-dependent accumulation of NQO1 in AD patients [35]. Expression of NQO1, which is a flavoenzyme that plays an important role in maintaining the cellular redox state, is induced in response to electrophilic and/or oxidative stress caused by exposure to chemical or endogenous quinones [31]. Like SOD3, the basal NQO1 expression varies with species, tissue, cell type, or aging phase. In the lung, liver, cerebellum, memory T cells, and retinal pigment epithelium, NQO1 expression is upregulated, while expression is downregulated in the astrocytes of aged mice [31]. In the current study, we demonstrated the overexpression of NQO1 in rhesus hippocampal aging. Recent studies have also shown that SLC7A11 overexpression inhibits ROS-induced ferroptosis and leads to a significant abrogation of the tumor suppression function of p53^3KR^, which can mediate senescence, apoptosis, and cell-cycle arrest [36, 37]. As a component of the NRF2 pathway, G6PD protects against endogenous oxidative DNA damage and neurodegeneration and prolongs the lifespan in aged mice [38, 39]. In this study, we demonstrated that G6PD, NQO1, PRDX6 SLC7A11, NQO1 and SOD3 were upregulated in rhesus hippocampus, which suggested strong antioxidant potential during the aging process in the hippocampus of rhesus monkeys.

Previous studies revealed a remarkable association of focal adhesion, PI3K–AKT, and mTOR signaling with age-related disease and longevity [40–43]. In the current study, we found focal adhesion PI3K–AKT–mTOR signaling was activated during hippocampal aging. Focal adhesion sites, which form the connection of cells with the ECM, consist of adhesion proteins such as integrins and extracellular proteins including fibronectin, laminin, vitronectin, and collagens) [40]. As the upstream extracellular components of focal adhesion signaling, collagens are progressively stabilized with age [44]. It has also been reported that collagenase-resistant Col1a1 promoted mouse aging and induced senescence of vascular smooth muscle cells (SMCs) [45]. Our results also showed marked upregulation of collagens, such as COL1A1, COL1A2, COL6A3, COL4A1, during rhesus hippocampal aging. In addition, caveolin-1 and integrins play critical roles in determining senescent cell morphology [46]. Recently, it was also reported that premature senescence induced by cavelin-1-deficiency is mediated by inhibition of mitochondrial respiration and oxidative phosphorylation complex I, a reduction in the NAD+/NADH ratio, and SIRT1 inactivation [47]. Activation of focal adhesion signaling induces PI3K–AKT signaling. This pathway is critically involved in regulation of the physiological responses associated with aging and a reduction in PI3K–AKT signaling has been reported to extend longevity across a wide range of species [48, 49]. AKT signaling predominantly induces anabolic, synthetic and growth systems via a complex network of targets, including mTOR, GSK3β, and FoxO [41]. The PI3K–AKT–GSK3β pathway, PI3K–AKT–FoxO axis, and PI3K–AKT–mTOR signaling pathway play significant roles in aging and age-related disease; however, PI3K–AKT–mTOR signaling was suggested as the most important modulator of aging and age-related disease [42, 50, 51]. mTOR, including mTORC1 and mTORC2, were crucial in controlling long-term synaptic efficacy and memory storage [52]. Long-term memory is influenced by the mTOR signaling pathway, with mTORC2 reported to control the actin polymerization required for consolidation [53, 54]. It is also reported that rapamycin-mediated inhibition of mTOR, a central regulator of protein synthesis in neurons, impairs the formation and reconsolidation of inhibitory avoidance memory in the hippocampus [55]. TSC1/2 possesses Rheb–GAP activity that directly antagonizes Rheb–mTOR signaling [56]. TSC1 upregulation inhibits PI3K–AKT signaling. It was reported that TSC1 overexpression extends lifespan in *D. melanogaster* [57]. Furthermore, Tsc1(+/−) mice exhibit a variety deficits in hippocampus-dependent learning and memory combined with impaired social behavior [58]. In the current study, RHEB expression in the hippocampus of rhesus monkeys was clearly downregulated. The decrease in RHEB-1 and mTOR signaling increases the lifespan of worm through inhibition of protein synthesis [59], a process that involves direct phosphorylation of the 4E-BP family of translational regulators (EIF4E and EIF4E2) and 70-kDa ribosomal S6 protein kinase (S6K1/2) by mTORC1 [60–62]. In neurons, EIF4E expression regulates the synthesis of several proteins involved in synaptic plasticity and memory formation [63]. Neurons require tight regulation of protein synthesis and degradation for maintenance of highly dynamic cellular processes, such as cell growth, synaptic formation, or synaptic plasticity and memory [64]. In our study, we observed marked downregulation of EIF4E and EIF4E2. Thus, the variations in focal adhesion PI3K–AKT–mTOR signaling indicate an important function in the rhesus hippocampus aging process.

Age-related changes in the protein synthesis machinery have revealed a decline in the efficiency and accuracy of ribosomal function [65]. In our study, marked downregulation of ribosome protein expression was detected during the hippocampus aging process, which may result in decreased protein synthesis. Furthermore, inhibition of protein synthesis extends lifespan in multiple species [60]. Considerable evidence suggests that the formation of long-term memories requires a critical period of new protein synthesis. The vital role of dendritic protein synthesis in long-term synaptic plasticity indicates the involvement of local translational control in memory processing [66]. To sum up, our evidence indicates that the marked downregulation of ribosomal protein expression may inhibit the aging process in rhesus hippocampus, but may affect the formation of long-term memories.

## Conclusion

In this study, we conducted a proteomics analysis of hippocampal during the aging in rhesus monkeys. Taken together, our results suggest the existence of age-related variations in rhesus hippocampal proteome with DEPs predominantly enriched in the ETC, NRF2 pathway, focal adhesion PI3K–AKT–mTOR signaling pathway, and cytoplasmic ribosome proteins. Overall, the data presented here provide a comprehensive protein profile of hippocampus of young and old rhesus monkeys (aged 6 and 20 years, respectively), in addition to shedding light on alterations in hippocampal protein expression during the aging process in this species.

The main limitation of this study was the small sample size (only 3 hippocampus sample in each group). In the future, the proteomic changes in the hippocampus of rhesus monkeys with increased sample size should be performed. The left–right asymmetry and differences between rhesus and human hippocampal aging also remain to be investigated. In addition, genomics, epigenomics, transcriptomics, and metabonomics studies are required to elucidate the mechanism, markers and potential therapeutic targets of hippocampal aging in rhesus monkeys and humans.

## Methods

### Hippocampus samples

Right hippocampus samples from two groups rhesus macaques (Group A: 6.01 ± 0.60 years, 1 male and 2 female; Group B: 19.97 ± 0.66 years, 2 male and 1 female) were obtained from the Institute of Medical Biology at the Chinese Academy of Medical Sciences and Peking Union Medical College. As reported, behavioral studies of verbal/spatial areas, affective domain, and sensorimotor processing of the aging brain demonstrated that the right hemisphere of the brain is more susceptible to aging than the left hemisphere [67]. Thus, investigations of aging of the right hippocampus are warranted. The current study was approved by the Ethical Review Committee of the Chinese Academy of Medical Sciences.

### Reagents

Reagents and kits were purchased from commercial sources as follows: iodoacetamide (IAA), dithiothreitol (DTT), and urea (GE Healthcare, Little Chalfont, Bucks, UK); proteinase inhibitor cocktail tablet mini (Roche, BS, CH); TMT™ Mass Tagging Kits (Thermo Fisher Scientific, NJ, USA); sequencing-grade endoproteinase trypsin/Lys-C mix (Promega, WI, USA), trifluoroacetic acid (TFA, Sigma). Furthermore, an Xbridge BEH300 C18 column (4.6 × 250 mm, 5 μm) was purchased from Waters (Milford, MA, USA). A fused silica capillary column (75 μm ID, 150 mm length) was obtained from Upchurch (Oak Harbor, WA, USA), and C18 resin (300 Å, 5 μm) was purchased from Varian (Palo Alto, CA, USA).

### Sample preparation

Proteins were extracted from the right hippocampus of rhesus macaques using of the liquid nitrogen homogenization method [14]. Tissues were homogenized in ice-cold lysis buffer (8 M urea and 1× cocktail in PBS, pH 8.0) and then transferred to a 1.5-mL tube on ice for centrifugation at 12,000 rpm (15 min, 4 °C) to remove cellular debris. Subsequently, the supernatant was transferred to a fresh 1.5-mL tube. Protein concentrations of the supernatants were determined using the NanoDrop 2000 (Thermo Scientific, NJ, USA) according to the manufacturer’s instructions. Supernatants from each group were combined in equal amounts. Supernatants were immediately stored at − 80 °C.

### TMT-labeling

For proteolytic digestion, 100 μg proteins from each mixed sample of the two groups in 8 M urea were alkylated with 10 mM DTT (1 h, 37 °C) and 25 mM IAA (30 min in the dark at room temperature). The concentration of urea was reduced from 8 to 1 M, which is required for trypsin/Lys-C activity, by dilution with PBS (pH 8.0). Trypsin/Lys-C mix (1:100 w/w) was then added into the reaction tube to digest the proteins to peptides (overnight at 37 °C) according to the manufacturer’s protocol. After digestion, the reaction was quenched by heating at 60 °C (30 min). Digested proteins were desalted, dried and finally dissolved in 50 μL 200 mM triethylammonium bicarbonate buffer (pH 8.5). Peptides of rhesus macaques were subjected to TMT-labeling using TMT-labeling Kit according to the manufacturer’s protocol. Different TMT tags were used to label the hippocampus samples from the two groups: TMT129 for the macaques aged 6 years (Group A) and TMT131 for the macaques aged 20 years (Group B). After labeling, the two labeled samples were pooled, desalted, dried and finally dissolved in 100 μL of 0.1% TFA.

### High-performance liquid chromatography separation (HPLC) fractionation

HPLC (UltiMate 3000 UHPLC, Thermo Scientific) was used to fractionate the TMT-labeled peptides (100 μL in 0.1% TFA) using an Xbridge BEH300 C18 column maintained at 45 °C with a flow rate of 1.0 mL/min. Peptides in the fractions were detected by ultraviolet absorbance at 214 nm. In this study, phase A was ddH_2_O (pH 10.0) and phase B was 98% acetonitrile (pH 10.0). The fractions (1.5 mL/tube) were collected, dried under vacuum and dissolved in 20 μL 0.1% TFA for LC–MS/MS analysis.

### Peptide analysis by LC–MS/MS

The TMT-labeled peptides were separated by a 135-min gradient elution procedure (flow rate, 0.3 μL/min) using the Ultimate U3000 system (Thermo Scientific, NJ, USA) fitted with a homemade fused silica capillary column (75 μm ID, 150 mm length; Upchurch) packed with C-18 resin (300 Å, 2 μm; Varian). The eluates were then analyzed with a directly interfaced Thermo Orbitrap Fusion Lumos mass spectrometer (Thermo Scientific, NJ, USA). In this study, mobile phase A was 0.1% formic acid and mobile phase B was 100% acetonitrile and 0.1% formic acid. Based on 3-s data-dependent MS/MS scans, a single full-scan mass spectrum was obtained in the Orbitrap (350–1550 m/z, 120,000 resolution), in which an Ion Routing Multipole was set at 35% normalized collision energy (HCD). Data collection was performed in the data-dependent acquisition mode. The MS/MS spectra from each LC–MS/MS run were searched against the selected Rhesus FASTA database from UniProt (unreviewed, released on November 15, 2017) using Proteome Discoverer 2.2 software (Thermo Scientific). According to the recommended software settings, the search criteria were set as follows: false discovery rate (FDR) = 0.01; full tryptic specificity was required; no more than two missed cleavages; carbamidomethylation (C, + 57.021 Da) and TMT-plex (lysine [K] and any N-terminal) were set as the static modifications; oxidation (methionine, M) was set as the dynamic modification; precursor ion mass (all MS from Orbitrap mass analyzer) tolerances were set at 20 ppm; the fragment ion mass tolerance was set at 20 mmu for all MS2 spectra acquired. The intensities of TMT-labeling per peptide were used for relative quantification of proteins according to manufacturer’s instructions. The ratios of altered proteins were set as TMT131/TMT129. The upregulation and downregulation thresholds were set as ≥ 1.2 for upregulation and ≤ 0.83 for downregulation.

### Bioinformatics analysis

The homologous analysis was conducted using the online db2db tool of bioDBnet (biological DataBase network, https://biodbnet-abcc.ncifcrf.gov/db/db2db.php). The enrichment analysis of high-confidence proteins was conducted by DAVID 6.8 (https://david.ncifcrf.gov/) and plotted using GraphPad Prism 7. To detect the protein–protein interaction network, STRING was performed using Cytscape 3.7.1 with STRING protein query as data source, in which the medium confidence threshold was set at 0.4, and the maximum additional interactors were set at 0. Furthermore, the online WEB-based GEne SeT analysis Toolkit (WebGestalt, Vanderbilt University, Nashville, TN, USA, http://www.webgestalt.org/option.php) was used for the gene ontology (GO) enrichment analysis and Wiki pathway enrichment analysis of DEPs. Cytoscape was used to visualize the differentially expressed proteins in matched pathways. Target pathways were selected from those fulfilling the following criteria: *p* < 0.05, with at least three target genes mapped. Adobe Illustrator CS5 and Adobe Photoshop CS4 were used for figure generation.

The mass spectrometry proteomics data have been deposited to the ProteomeXchange Consortium via the PRIDE [68] partner repository with the dataset identifier PXD011398.

### Western blot

Protein concentrations were determined using a NanoDrop 2000 (Thermo Scientific, NJ, USA). Twenty microgram of tissue lysates in each sample was loaded into polyacrylamide gels for SDS-PAGE, and transferred onto nitrocellulose membranes. The membranes were then blocked with 5% non-fat dry milk in TBS-T (Tris Buffered Saline plus 0.5% Tween) for 60 min and incubated with primary antibodies (ACTB, GTX124213, GeneTex; RPL23, 16086-1-AP, Proteintech; GSTM3, ab74749, Abcam) overnight at 4 °C. Horseradish peroxidase-conjugated secondary antibodies were used to visualize protein bands. Band intensity was analyzed after incubation with ECL reagents, and imaged.

## Supplementary information

**Additional file 1: Table S1.** All differentially expressed proteins detected.

**Additional file 2: Table S2.** Wiki pathway analysis of DEPs.

**Additional file 3: Figure S1.** Similarities in binding sequences of antibodies.

## Data Availability

All data generated or analyzed during this study are included in this published article and its additional information files.

## Abbreviations

DDT: dithiothreitol
DEPs: differential expressed proteins
ETC: the electron transport chain
GO: gene ontology
HPLC: high-performance liquid chromatography
IAA: iodoacetamide
LC–MS/MS: liquid chromatography-tandem mass spectrometry
TMT: tandem mass tags
NRF2: the nuclear factor erythroid 2-related factor 2
PI3K–AKT–mTOR: phosphatidylinositol 3-kinase/protein kinase/serine/threonine-protein kinase mTOR
ATP: adenosine triphosphate
ROS: reactive oxygen species
NQO1: NAD(P)H dehydrogenase [quinone] 1 isoform a
PRDX6: peroxiredoxin-6
SOD3: superoxide dismutase [Cu–Zn]
G6PD: glucose-6-phosphate 1-dehydrogenase
GSH: glutathione
ECM: extracellular matrix
COL1A1: collagen alpha-1(I) chain preproprotein
COL1A2: collagen alpha-2(I) chain
COL6A3: collagen type VI alpha 3 chain
COL4A1: collagen type IV alpha 1 chain
NAD+/NADH: nicotinamide adenine dinucleotide
GSK3β: glycogen synthase kinase 3
FoxO: forkhead box O
mTORC1: mTOR complex 1
mTORC2: mTOR complex 2
Rheb–GAP: GTP-binding protein Rheb/GTPase-activating protein
TSC1/2: tuberous sclerosis ½
RHEB-1: GTP-binding protein RHEB1
EIF4E: eukaryotic translation initiation factor 4E
EIF4E2: eukaryotic translation initiation factor 4E2
RPL: 60S ribosomal protein
RPS: 40S ribosomal protein S3
NDUFA: NADH dehydrogenase [ubiquinone] 1 alpha subcomplex subunit
NDUFB: NADH dehydrogenase [ubiquinone] 1 beta subcomplex subunit
NDUFS: NADH dehydrogenase [ubiquinone] iron–sulfur protein
FTH1: ferritin 1
FTL: ferritin
CES1: carboxylic ester hydrolase 1
SLC2A1: solute carrier family 2 member 1
SLC5A3: sodium/myo-inositol cotransporter
GSTM3: glutathione *S*-transferase 3
GSTM5: glutathione *S*-transferase 5
ErbB: receptor tyrosine kinase
ITGB1: integrin subunit beta 1
ITGB8: integrin subunit beta 8
CAV1: caveolin-1
