# Supplementary material for: Proteomics profiling and pathway analysis of hippocampal aging in rhesus monkeys
Source: BMC Neurosci. 2020 Jan 15;21:2. doi: 10.1186/s12868-020-0550-4 (PMC6964096; doi:10.1186/s12868-020-0550-4)
Supplement: Supplementary file 2 — Additional file 2: Table S2. Wiki pathway analysis of DEPs. [file 12868_2020_550_MOESM2_ESM.docx]

| Table S2. Wiki pathway analysis of DEPs. | | | | | |
| --- | --- | --- | --- | --- | --- |
| Pathway | Gene Symbol | Gene Name | Entrez Gene ID | *P*-Value | FDR |
| Focal Adhesion-PI3K-Akt-mTOR-signaling pathway | LAMC3, COL1A1, COL1A2, COL4A1, EGFR, EIF4E, LAMA1, KIT, PIK3C2A, RHEB, SLC2A1, TSC1, IKBKG | laminin subunit gamma 3, collagen type I alpha 1 chain, collagen type I alpha 2 chain, collagen type IV alpha 1 chain, epidermal growth factor receptor, eukaryotic translation initiation factor 4E, laminin subunit alpha 1, KIT proto-oncogene receptor tyrosine kinase, phosphatidylinositol-4-phosphate 3-kinase catalytic subunit type 2 alpha, Ras homolog enriched in brain, solute carrier family 2 member 1, tuberous sclerosis 1, inhibitor of nuclear factor kappa B kinase subunit gamma | 10319, 1277, 1278, 1282, 1956, 1977, 284217, 3815, 5286, 6009, 6513, 7248, 8517 | 0.0397 | 0.68 |
| Focal Adhesion | LAMC3, COL1A1, COL1A2, COL4A1, EGFR, FLNA, LAMA1, PPP1R12C, PRKCB, MAPK8, VASP, CAV1 | laminin subunit gamma 3, collagen type I alpha 1 chain, collagen type I alpha 2 chain, collagen type IV alpha 1 chain, epidermal growth factor receptor, filamin A, laminin subunit alpha 1, protein phosphatase 1 regulatory subunit 12C, protein kinase C beta, mitogen-activated protein kinase 8, vasodilator-stimulated phosphoprotein, caveolin 1 | 10319, 1277, 1278, 1282, 1956, 2316, 284217, 54776, 5579, 5599, 7408, 857 | 0.00683 | 0.621 |
| Insulin Signaling | EIF4E, GAB1, PIK3C2A, PRKCB, MAPK8, RHEB, SLC2A1, SNAP25, TSC1, SNAP23 | eukaryotic translation initiation factor 4E, GRB2 associated binding protein 1, phosphatidylinositol-4-phosphate 3-kinase catalytic subunit type 2 alpha, protein kinase C beta, mitogen-activated protein kinase 8, Ras homolog enriched in brain, solute carrier family 2 member 1, synaptosome associated protein 25, tuberous sclerosis 1, synaptosome associated protein 23 | 1977, 2549, 5286, 5579, 5599, 6009, 6513, 6616, 7248, 8773 | 0.00707 | 0.621 |
| Cytoplasmic Ribosomal | RPL10A, RPL4, RPL13, RPL22, RPL23A, RPL24, RPL26, RPL27, RPL27A, RPL35A, RPLP1, RPS4Y1, RPS9, RPS23, RPL23 | ribosomal protein L10a, ribosomal protein L4, ribosomal protein L13, ribosomal protein L22, ribosomal protein L23a, ribosomal protein L24, ribosomal protein L26, ribosomal protein L27, ribosomal protein L27a, ribosomal protein L35a, ribosomal protein lateral stalk subunit P1, ribosomal protein S4, Y-linked 1, ribosomal protein S9, ribosomal protein S23, ribosomal protein L23 | 4736, 6124, 6137, 6146, 6147, 6152, 6154, 6155, 6157, 6165, 6176, 6192, 6203, 6228, 9349 | 5.4E-09 | 2.23E-06 |
| NRF2 pathway | CES1, NQO1, SLC7A11, FTH1, FTL, GSTM3, GSTM5, SLC2A1, SOD3 | carboxylesterase 1, NAD(P)H quinone dehydrogenase 1, solute carrier family 7 member 11, ferritin heavy chain 1, ferritin light chain, glutathione S-transferase mu 3, glutathione S-transferase mu 5, solute carrier family 2 member 1, superoxide dismutase 3 | 1066, 1728, 23657, 2495, 2512, 2947, 2949, 6513, 6649 | 0.0105 | 0.621 |
| Ras Signaling | EGFR, GAB1, KIT, PLD2, PRKCB, MAPK8, TIAM1, IKBKG | epidermal growth factor receptor, GRB2 associated binding protein 1, KIT proto-oncogene receptor tyrosine kinase, phospholipase D2, protein kinase C beta, mitogen-activated protein kinase 8, T-cell lymphoma invasion and metastasis 1, inhibitor of nuclear factor kappa B kinase subunit gamma | 1956, 2549, 3815 5338, 5579, 5599, 7074, 8517 | 9.44e-02 | 7.14e-01 |
| Target Of Rapamycin (TOR) Signaling | RICTOR, RHEB, TSC1 | RPTOR independent companion of MTOR complex 2, Ras homolog enriched in brain, tuberous sclerosis 1 | 253260, 6009, 7248 | 0.0663 | 0.68 |
| Electron Transport Chain | NDUFA4, NDUFA10, NDUFB7 | NDUFA4 mitochondrial complex associated, NADH:ubiquinone oxidoreductase subunit A10, NADH:ubiquinone oxidoreductase subunit B7 | 4697, 4705, 4713 | 0.0406 | 1 |
| ATM Signaling Pathway | RAD50, MRE11, IKBKG | RAD50 double strand break repair protein, MRE11 homolog, double strand break repair nuclease, inhibitor of nuclear factor kappa B kinase subunit gamma | 10111, 4361, 8517 | 0.08 | 0.68 |
