# Supplementary figures and images for: Proteomics profiling and pathway analysis of hippocampal aging in rhesus monkeys
Source: BMC Neurosci. 2020 Jan 15;21:2. doi: 10.1186/s12868-020-0550-4 (PMC6964096; doi:10.1186/s12868-020-0550-4)

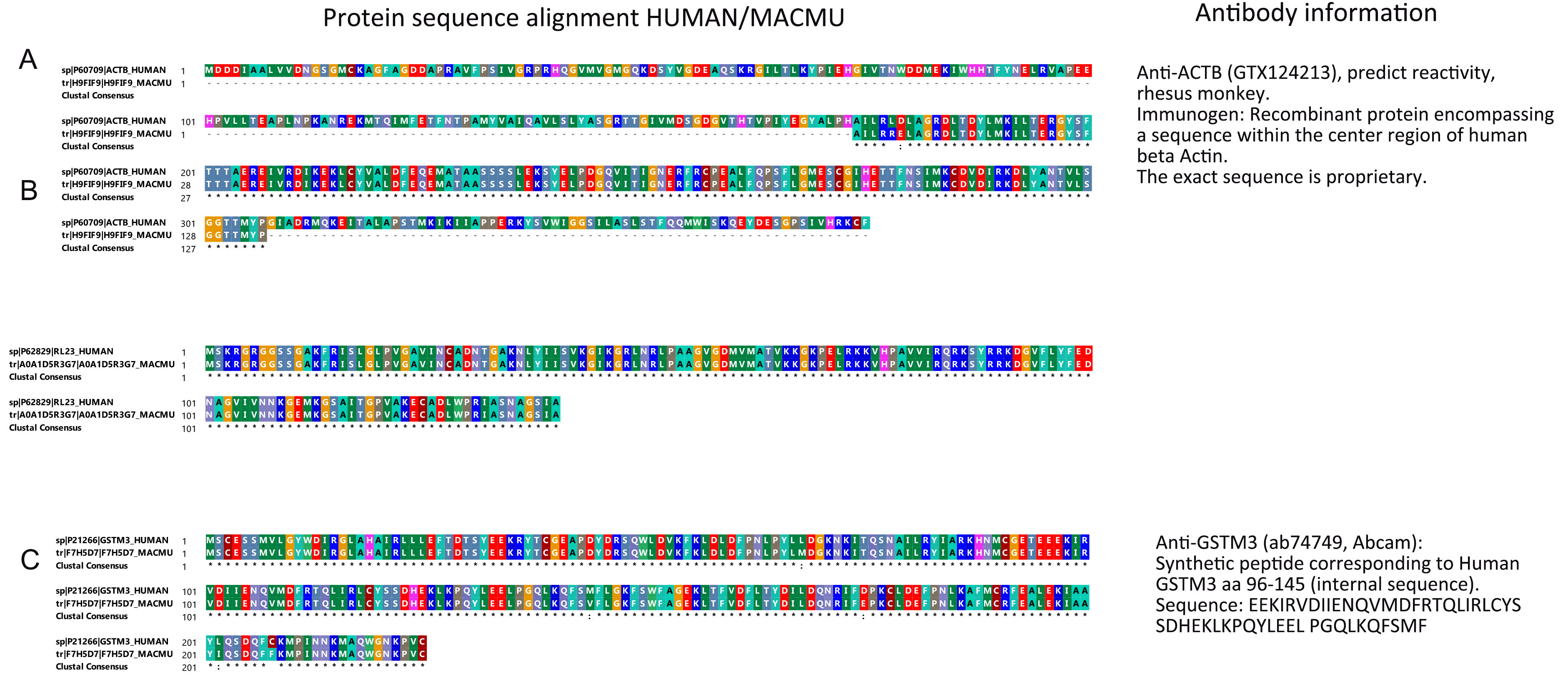

Supplement: Supplementary file 3 — Additional file 3: Figure S1. Similarities in binding sequences of antibodies. [file 12868_2020_550_MOESM3_ESM.jpg]
